# Outcomes of Immune Thrombotic Thrombocytopenic Purpura (iTTP) With Upfront Cyclophosphamide vs. Rituximab

**DOI:** 10.3389/fmed.2020.588526

**Published:** 2020-10-28

**Authors:** Mouhamed Yazan Abou-Ismail, Yasmin Arafah, Pingfu Fu, Shufen Cao, Alvin H. Schmaier, Lalitha Nayak

**Affiliations:** ^1^Case Western Reserve University, Cleveland, OH, United States; ^2^Division of Hematology and Oncology, Department of Internal Medicine, University Hospitals Cleveland Medical Center, Cleveland, OH, United States

**Keywords:** thrombotic thrombocytopenic purpura, rituximab, cyclophosphamide, treatment, relapse

## Abstract

**Background:** Immune thrombotic thrombocytopenic purpura (iTTP) is a rare, life-threatening disorder managed with plasma exchange (PLEX) and steroids. Addition of rituximab (RTX) to initial disease treatment has been shown to lower future relapse rates. Information as to whether upfront cyclophosphamide (CTX) treatment is helpful in reducing relapse is not known.

**Methods:** In a retrospective cohort study, we identified all patients at our institution diagnosed with iTTP between 2010 and 2019. We analyzed outcomes of cumulative incidence of relapse (CIR) and duration of remission.

**Results:** Thirty Nine patients were studied. Group A (*n* = 10) included patients who received upfront PLEX and steroids alone, and Group B (*n* = 28) included those who received either upfront RTX (*n* = 23) or CTX (*n* = 5) in addition to PLEX and steroids. The 2-year CIR was 50% in Group A and 27.7% in Group B, with a median duration of remission of 43.6 months vs. 108.3 months, respectively (*p* = 0.04). Group A was associated with a HR=8.7 (95% CI: 1.27, 59.45), *p* = 0.027 for duration of remission. There was no significant difference between CTX and RTX in both outcomes of CIR and duration of remission. We observed a potential impact on remission duration based on the presenting absolute neutrophil count (HR = 0.74, 95% CI: 0.58, 0.96) and serum creatinine (HR = 1.42, 95% CI: 1.03, 1.94).

**Conclusion:** There was no significant difference in iTTP relapse outcomes between upfront RTX and CTX. Absolute neutrophil count and serum creatinine may have a role in predicting relapse. Larger, prospective studies are needed to evaluate these findings.

## Introduction

Auto-immune thrombotic thrombocytopenic purpura (iTTP) is a rare, life-threatening disorder caused by auto-antibodies against ADAMTS13. It is characterized by a severe thrombotic microangiopathy (TMA) that leads to organ failure and is associated with high morbidity and mortality. The mortality rate of the untreated disease is around 90%, and treatment with corticosteroids and therapeutic plasma exchange (PLEX) reduces that rate to around 10%. (1, 2) Although this treatment induces remission, disease relapse remains a common problem. Relapse is estimated to occur in around 30–50% of patients after achieving initial remission (3, 4). Since iTTP is mediated by an antibody against ADAMTS13, additional immunosuppressive therapy given upfront has been shown to lower relapse rates (5–9).

Rituximab (RTX) is a monoclonal antibody that targets B-cells, which produce the antibody responsible for causing iTTP. It was first used in treating this disease in the relapsed or refractory setting in the early 2000s, and was successful in inducing remission (10, 11). The Phase II trials by Scully et al. and Chen et al. demonstrated safety and efficacy of using upfront RTX (8, 12). In the former trial, RTX was associated with 10% relapse rate compared to 57% in historical controls, which was a statistically significant reduction (8). It has since been used in the upfront setting in various studies and shown to lower relapse rates (5–9). While recent common practice has shifted toward adding RTX to steroids and PLEX as front-line treatment for acute initial iTTP, this practice has not been rigorously examined.

An alternative therapy, cyclophosphamide (CTX), has been used in relapsed iTTP. CTX is effectively used in the management of acquired hemophilia that is caused by auto-antibodies to factor VIII (13, 14). In the European Acquired Hemophilia Registry, steroids combined with cyclophosphamide resulted in more stable complete remission (70%) than rituximab-based regimens (59%) (14). Several small studies have utilized cyclophosphamide in the treatment of relapsed or refractory iTTP where it has been shown to be effective (15–19). However, studies evaluating CTX use in the upfront setting as a first-line treatment along with PLEX and corticosteroids are lacking. There are several advantages to the use of CTX. First, many patients develop infusion reactions to RTX or have other contraindicating comorbidities that may preclude its use upfront. Furthermore, if given concurrently with PLEX, ~65% of RTX may be removed making the optimal dose and frequency of this drug difficult to determine (20). Alternatively, CTX is unlikely to be removed by PLEX due to its low protein-binding rate of 23%, and higher volume of distribution of 0.8 L/kg (21). Furthermore, CTX is far less costly than RTX. As such, having an alternative agent for use in the upfront setting may be beneficial. In this retrospective clinical review, we hypothesized that CTX is as effective as RTX in lowering relapse rate when used in the upfront setting for initial acute iTTP. We compared outcomes of cumulative incidence of relapse (CIR) as well as duration of remission between patients who received upfront CTX and those who received RTX, in addition to PLEX and steroids. Subsequently, in order to evaluate whether there was benefit in the use of any additional immunosuppression besides PLEX and steroids, we compared the cohort of patients who received either additional CTX or RTX with those who received PLEX and steroids alone without any additional immunosuppression. This retrospective investigation also examined several baseline characteristics on initial disease presentation and assessed whether they correlated with a higher risk of relapse.

## Methods

### Patient Selection and Data Collection

In a retrospective chart review, we identified all patients diagnosed with iTTP at University Hospitals—Cleveland Medical Center between 2010 and 2019. The study was approved by the Institutional Review Board (IRB). A billing diagnosis code search for “thrombotic microangiopathy” (ICD9: 446.6, ICD10: M31.1) was conducted. We included all patients diagnosed with iTTP, defined as having an ADAMTS13 level of <10% and a positive ADAMTS13 inhibitor or antibody, with clinical evidence of TMA per provider documentation. We excluded patients with a follow-up duration of <30 days, or with TMA due to other causes such as congenital TTP, hemolytic-uremic syndrome, anti-phospholipid syndrome, HELLP syndrome, systemic lupus erythematosus, preeclampsia, accelerated hypertension, and diffuse intravascular coagulopathy. The ADAMTS13 assays were performed at the Blood Center of Wisconsin, measured by Fluorescence Resonance Energy Transfer with a synthetic substrate. Inhibitor assays were performed on those patients with low ADAMTS13 levels.

**Figure 1:**
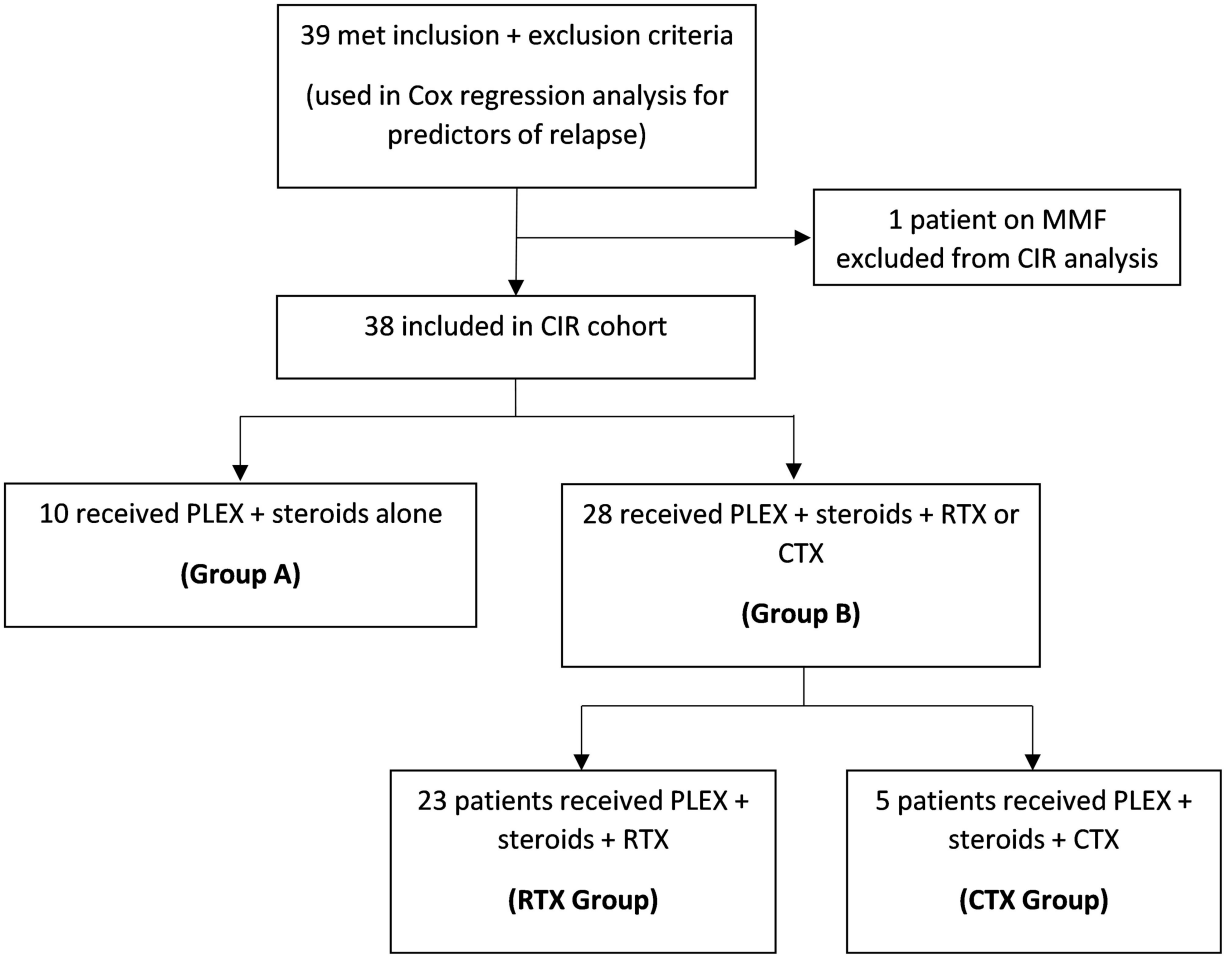
The four different groups used for CIR analysis.

We divided our cohort into four groups (Figure 1). Group A included patients who received steroids and PLEX alone, without any additional upfront therapy. Group B included patients who received steroids, PLEX, in addition to either RTX or CTX. The latter group was further divided into RTX group and CTX group to compare the effects of these two agents. For our secondary objective of predictors of relapse, we utilized the collective data from the entire cohort (all groups combined).

### Outcomes and Definitions

We defined the “onset of remission” as the date after which the platelet count was greater than or equal to 150,000 /μl and lactate dehydrogenase (LDH) ≤ 246 U/L for a minimum of 48 h. Clinical relapse was defined as recurrence of TMA in addition to ADAMTS13 level of <10%. The “duration of remission” was measured from the date of onset of remission, until the date of first clinical relapse of TTP. For patients who are alive and did not relapse, the end point was the date of last-follow-up.

### Statistical Methods

The cumulative incidence of relapse (CIR) was estimated using Kaplan-Meier method (22) and its difference among treatment groups was examined by log-rank test. The effect of continuous and categorical covariates on thrombotic thrombocytopenic purpura relapse rate was estimated by univariate Cox model (23). The association of categorical variables and continuous variables was examined using chi-square test and Pearson correlation coefficient, respectively, and the difference of continuous measurements among groups was tested using *T*-test (two groups) or ANOVA (more than 2 groups). The effect of continuous and categorical covariates on time to eradication of inhibitor (from diagnosis time) was estimated by univariate and multivariable linear regression. The effect of continuous and categorical covariates on days of hospitalization duration was also estimated by univariate and multivariable linear regression. All tests are two-sided and *p* ≤ 0.05 was considered statistically significant.

## Results

A total of 39 patients met the inclusion and exclusion criteria. One patient received mycophenolate mofetil, and was not included in the primary analysis of CIR, but was included in the secondary objective of the study on laboratory predictors of relapse. Ten patients received steroids and PLEX alone (Group A), and 28 patients received steroids, PLEX, and either RTX or CTX (Group B). Of those, 23 received RTX (375 mg/m^2^ weekly for four doses), and five received CTX (400 mg/m^2^ every 3 weeks for six doses). The average age of diagnosis was 44 years. The average duration of follow-up was 76 months. There were no significant differences in baseline characteristics between any of the groups (Table 1). Upfront administration of CTX was not associated with any significant side effects in the five treated patients.

**Table 1:** Baseline Characteristics.

**Variables**	**CTX group (*n* = 5) mean** **(STD) or frequency**	**RTX group (*n* = 23) mean** **(STD) or frequency**	**Group A (*n* = 10) mean** **(STD) or frequency**	***p*-value**
Age (years)	33 (7.80)	44 (20.55)	43 (13.4)	0.44
Gender (F/M)	5/0	13/10	7/3	0.169
Initial inhibitor level	1.98 (2.50)	2.97 (2.72)	2.30 (2.51)	0.73
Initial ADAMTS13 antibody level	37.5 (34.65)	33.83 (29.08)	N/A	0.89
D-Dimer	767.00 (202.34)	2255.57 (1801.57)	1590.75 (600.06)	0.31
Fibrinogen	264.40 (67.42)	302.95 (90.46)	319.00 (117.66)	0.63
Nadir platelet count	8.8 (2.28)	12.5 (8.6)	12.20 (7.36)	0.64
Absolute neutrophil count	7.1 (4.72)	8.8 (4.1)	6.97 (2.14)	0.55
Absolute lymphocyte count	1.61 (1.06)	2.07 (1.05)	1.91 (1.40)	0.70
Neutrophil-to-lymphocyte ratio	9.59 (12.59)	6.23 (5.85)	5.61 (4.09)	0.61
Creatinine on admission	1.02 (0.22)	1.93 (1.98)	3.03 (1.47)	0.31
Peak LDH on admission	1,444.4 (790.47)	1,222.91 (604.31)	926.20 (374.53)	0.41
Platelet normalization (days)	11.2 (7.73)	11.32 (11.41)	11.20 (16.13)	0.99
LDH normalization (days)	11.6 (6.62)	11.33 (10.6)	6.00 (3.16)	0.58
Resolution time (days)	14.6 (8.32)	14.5 (12.07)	12.80 (15.15)	0.96
Duration of hospitalization (days)	23.6 (9.29)	26.75 (15.98)	11.2 (6.72)	0.13
Altered mental status (Yes/ No)	2/3	8/13	2/2	0.91
Autoimmune disease (Yes/No)	3/2	3/20	2/8	0.07
Malignancy (Yes/No)	3/2	4/19	1/9	0.06

### Clinical Relapse

**Figure 2:**
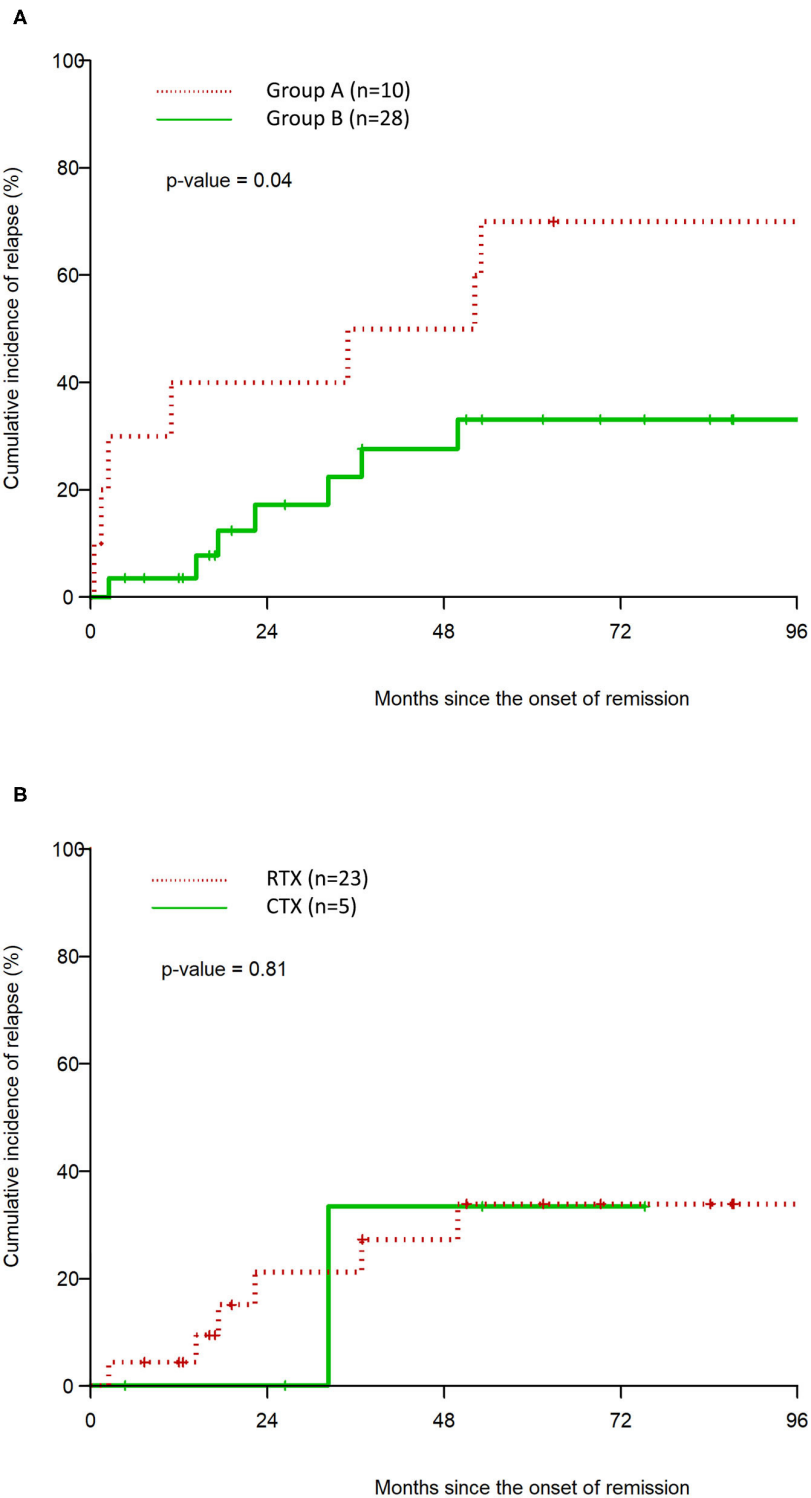
Kaplan–Meier estimation of cumulative incidence of clinical relapse after remission for Group A vs. Group B **(A)**, and CTX vs. RTX **(B)**.

The median duration of remission in Group A vs. Group B was 43.6 months (95% CI: 0.5, 114.3) and 108.3 months (95% CI: 36.8, N/A) respectively (*p* = 0.04). The CIR at 48 months in Group A was 50 vs. 27.7% (CTX = 33.3%, RTX = 27.2%) in Group B (Table 2) In the multivariable analysis, Group A was associated with a statistically significant hazard ratio of 8.7 (95% CI: 1.27, 59.45), *p* = 0.027 for time to clinical relapse, compared to Group B. There was no statistically significant difference in CIR between RTX and CTX in the Kaplan–Meier analysis (Figure 2, Table 2).

**Table 2:** Kaplan–Meier estimation of the cumulative incidence of clinical relapse (CIR, %).

**Factor**	**Cumulative incidence of relapse (CIR) for clinical relapse**				***p*-value**
	**24 months**	**48 months**	**72 months**	**120 months**	
Group A (*n* = 10)	40.0	50.0	70.0	85.0	0.04
Group B (*n* = 28)	17.2	27.7	33.2	66.6	
Cyclophosphamide (*n* = 5)	0.0	33.3	33.3		0.81
Rituximab (*n* = 23)	21.1	27.2	33.8	66.9	

### Predictors of Relapse

The Cox regression analysis of the effect of initial inhibitor level, absolute neutrophil count (ANC), nadir platelet count, and serum creatinine on the outcome of duration of remission is summarized below, and highlighted in Table 3. The analysis on all other variables, including age, absolute lymphocyte count, neutrophil/lymphocyte ratio, peak LDH on admission, fibrinogen, D-Dimer, presence of autoimmune disease, and presence of malignancy did not show a statistically significant impact on duration of remission. Similarly, the time to initiation of CTX or RTX from the time of disease diagnosis did not demonstrate an effect on the duration of remission.

**Table 3:** Univariate and multivariable Cox regression analysis on time to clinical relapse.

**Variable (per unit increase)**	**Clinical relapse HR (95% CI)**	
	**Univariate**	**Multivariable**
Initial inhibitor level	1.16 (0.94, 1.44), *p*-value: 0.17	1.23 (0.97, 1.56), *p*-value: 0.084*
Nadir platelet count	0.98 (0.90, 1.06), *p*-value: 0.60	
Initial ANC	0.79 (0.65, 0.98), *p*-value: 0.029	0.74 (0.58, 0.96), *p*-value: 0.020
Initial serum creatinine	1.36 (1.02, 1.82), *p*-value: 0.035	1.42 (1.03, 1.94), *p*-value: 0.032

* *Controlled for type of therapy only. All the other analyses controlled for all the baseline characteristics listed in Table 1*.

### Initial Inhibitor Level

Although the initial inhibitor level showed a trend toward an increased risk of clinical relapse per unit increase the results did not meet statistical significance in the univariate analysis (HR: 1.16, 95% CI: 0.95, 1.44), *p* = 0.17. However, this effect was amplified after controlling for type of upfront therapy in the multivariable analysis (HR: 1.23, 95% CI: 0.97, 1.56), *p* = 0.084.

### Absolute Neutrophil Count (ANC)

**Figure 3:**
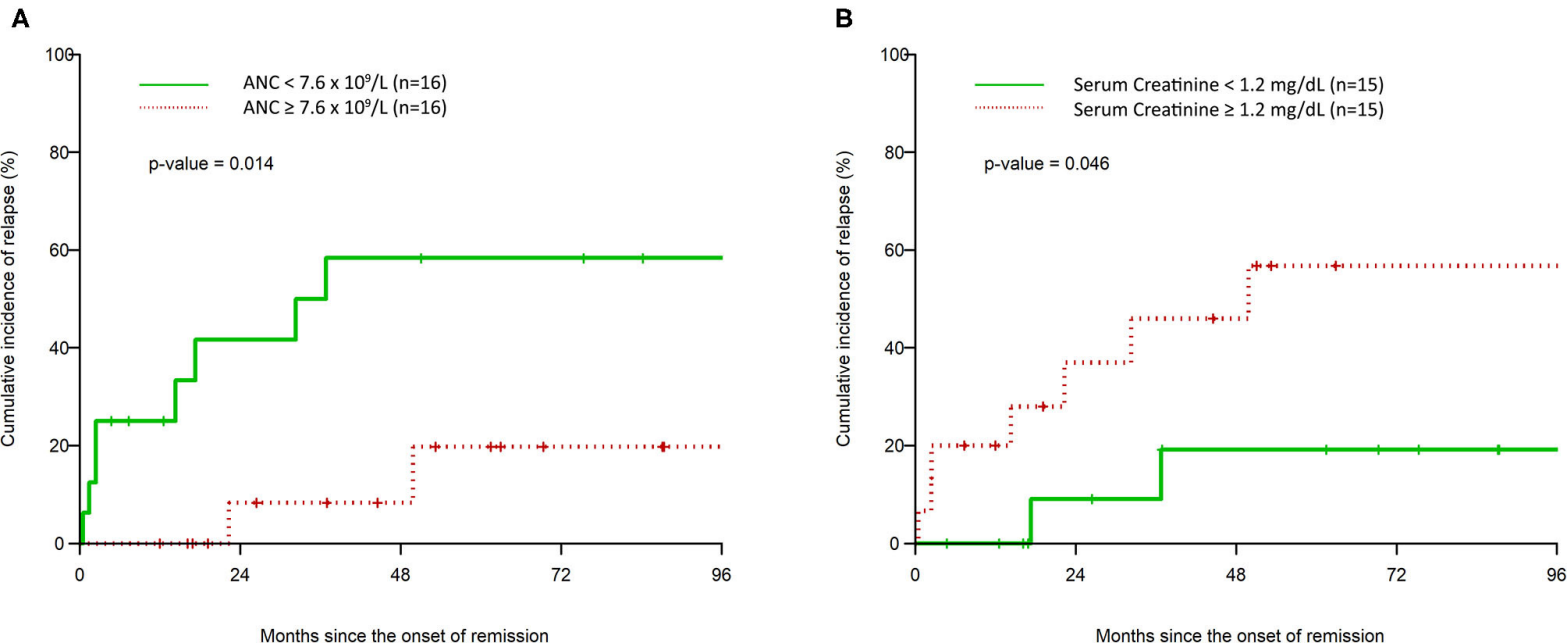
Kaplan–Meier estimation of cumulative incidence of clinical relapse after remission based on high vs. low initial absolute neutrophil count **(A)** and high vs. low initial serum creatinine **(B)**.

A higher ANC on presentation was associated with a statistically significantly longer duration of remission per unit increase. This effect maintained statistical significance in the multivariable analysis, with an HR of 0.74 (95% CI: 0.58, 0.96). Also, a Kaplan-Meier graph of a dichotomized separation of high or low ANC (above or below the median of 7.6 × 10^9^/L) shows a statistically significant decrease in the risk of CIR in the high ANC group (Figure 3A), with *p* = 0.015. Although the average ANC between the groups was not different, within the entire cohort there is a gradient of ANC values. Those ANC values >7.6 × 10^9^/L demonstrate the improved CIR finding.

### Nadir Platelet Count

The nadir platelet count on presentation was not associated with a statistically significant change in time to clinical relapse, with HR of 0.98 (0.90, 1.06), *p*-value: 0.60.

### Serum Creatinine

The serum creatinine level on presentation was associated with a decreased time to clinical relapse per unit increase, which was statistically significant in the multivariable analysis (HR: 1.42, 95% CI: 1.03, 1.94). A Kaplan–Meier graph of a dichotomized separation of high or low serum creatinine (above or below the median of 1.2 mg/dL) shows a statistically significant increase in the risk of CIR in the high serum creatinine group (Figure 3B), with *p* = 0.046.

## Discussion

Our study results demonstrate that the upfront use of immunosuppressive therapy, in addition to steroids and PLEX is associated with a reduction in the cumulative incidence of relapse and prolongs the duration of remission. The median duration of remission was significantly higher in Group B (108.3 months) compared to steroids and PLEX alone in Group A (43.6 months) with a HR of 8.7 (95% CI: 1.27, 59.45) after controlling for baseline characteristics in the multivariable model. Consistent with previously reported data, these findings demonstrate that the addition of immunosuppressive therapy is associated with a longer initial remission in iTTP. Our data is derived from a combined cohort of patients who received either CTX or RTX and demonstrates the benefit of any additional upfront immunosuppression besides PLEX and steroids in this disease. The sample size in the RTX and CTX groups individually was too small to demonstrate a statistically significant difference in outcomes in either group.

We then compared the outcomes between those who received RTX or CTX upfront. The use of upfront RTX has been shown in several studies to lower relapse rates (6–9). CTX, on the other hand, has not been studied in the upfront setting, as opposed to relapsed or refractory disease. While our sample size is small, the Kaplan–Meier curves showed clear overlap and no statistically significant changes in relapse outcomes in patients who received upfront CTX as compared to RTX. However, in order to achieve 80% power to detect a difference, the required total sample size is 308 patients. Nevertheless, our data demonstrate that it would be beneficial to evaluate the use of upfront CTX in larger, prospective studies. Low-dose pulse intravenous CTX used for iTTP is associated with far less adverse effects compared to higher doses of CTX, which can lead to infectious complications, bone marrow suppression, and long-term risk for malignancy (24). The risks with low-dose CTX are outweighed in the context of preventing relapse of life-threatening iTTP.

The second goal of our study was to investigate baseline characteristics that may help predict the risk of relapse. Identification of risk factors predictive of relapse would make a stronger argument for adding an upfront immunosuppressive agent, or using more aggressive therapy at diagnosis. A previous study by Tuncer et al. demonstrated that male sex, severe thrombocytopenia, and higher LDH pre-/post-treatment ratio were associated with higher risk of relapse (3). However, from all the different baseline characteristics we analyzed, we identified three different variables that may impact relapse outcomes: inhibitor level, ANC, and serum creatinine.

The inhibitor level on presentation did not show a statistically significant effect on duration of remission in the univariate analysis. However, when we controlled for the type of therapy used (Group A vs. Group B), its effect was amplified and trended closer toward statistical significance. This suggests that with larger study samples, this effect may be more pronounced. Of note, there are limitations in the inhibitor assay that we use since it does not report levels above 8 arbitrary units. Unlike previously reported data, our study did not demonstrate a statistically significant impact of nadir platelet count on relapse risk (3). Further, we found that a higher serum creatinine was associated with a shorter duration of remission and a statistically significant separation in the Kaplan–Meier analysis for CIR. We suspect that this may reflect a more aggressive nature of the disease and its microvascular complications, which may also translate into a higher tendency for clinical relapse.

We proposed that an elevated ANC may correlate with higher risk of relapse. However, contrary to our hypothesis, we found that a higher ANC was actually associated with a longer duration of remission. The protective effect of a higher ANC maintains statistical significance in the multivariable analysis after controlling for all other characteristics and is statistically significant in the Kaplan-Meier separation. At present, the mechanistic basis of this observation is not known. There is emerging evidence in the literature that neutrophils may inhibit B-cell responses, especially in the context of autoimmune diseases (25). In murine models, data suggest a role for neutrophils in suppressing immunoglobulin and antibody production in B lymphocytes, as well as slowing disease progression of murine lupus (26–28). There is also evidence that such an effect may occur in humans as well. A study by Lelis et al. has shown that myeloid derived suppressor cells, which may function as pathologically activated neutrophils, have a role in modulating B cell responses by suppressing B-cell proliferation and antibody production (29). Since iTTP is also an autoimmune disease caused by auto-antibodies against ADAMTS13, a modulatory effect on the B cell production of antibodies probably would correlate with a more durable remission.

## Conclusion

Our study is the first to examine outcomes in a subset of patients treated with upfront CTX therapy for initial iTTP, and suggests similar time to first relapse and 4-year CIR as compared to RTX. Although our numbers are small, our institutional experience suggests that CTX may be considered as alternative therapy in patients intolerant to RTX. Consistent with the currently published literature, PLEX and steroid therapy alone was associated with a significantly shorter duration of remission compared to additional immunosuppressive therapy. Initial inhibitor level, serum creatinine, and ANC may offer a predictive role in the risk for disease relapse. The results of these investigations indicate that there is room for more diverse approaches to the management of iTTP. Larger prospective studies are warranted to confirm these observations.

## Data Availability Statement

The raw data supporting the conclusions of this article will be made available by the authors, without undue reservation.

## Ethics Statement

Ethical review and approval was not required for the study on human participants in accordance with the local legislation and institutional requirements. Written informed consent for participation was not required for this study in accordance with the national legislation and the institutional requirements.

## Author Contributions

MA-I and YA collected the data. MA-I, AHS, and LN wrote the manuscript. AHS and LN supervised and mentored the study. All authors contributed to the article and approved the submitted version.

## Conflict of Interest

The authors declare that the research was conducted in the absence of any commercial or financial relationships that could be construed as a potential conflict of interest.
